# Impaction of regurgitation jet on anterior mitral leaflet is associated with diastolic dysfunction in patients with bicuspid aortic valve and mild insufficiency: a cardiovascular magnetic resonance study

**DOI:** 10.1007/s10554-021-02384-z

**Published:** 2021-08-26

**Authors:** Nicola Galea, Giacomo Pambianchi, Giulia Cundari, Francesco Sturla, Livia Marchitelli, Carolina Putotto, Paolo Versacci, Ruggero De Paulis, Marco Francone, Carlo Catalano

**Affiliations:** 1 Department of Experimental Medicine, “Sapienza” University of Rome, Viale del Policlinico 155, 00161 Rome, Italy; 2 Department of Radiological, Oncological and Pathological Sciences, “Sapienza” University of Rome, Rome, Italy; 3 Computer Simulation Laboratory, IRCCS Policlinico San Donato, San Donato Milanese (MI), Italy; 4 Department of Pediatrics, Obstetrics and Gynecology, “Sapienza” University of Rome, Rome, Italy; 5 Department of Cardiac Surgery, European Hospital, Rome, Italy; 6 Unicamillus International Medical University in Rome, Rome, Italy; 7 Department of Biomedical Sciences, Humanitas University, Via Rita Levi Montalcini 4, Pieve Emanuele, 20090 Milan, Italy; 8 IRCCS Humanitas Research Hospital, via Manzoni 56, Rozzano, 20089 Milan, Italy

**Keywords:** Bicuspid aortic valve, Regurgitation, Diastolic dysfunction, Myocardial strain, 4D flow imaging

## Abstract

To assess the impact of regurgitant jet direction on left ventricular function and intraventricular hemodynamics in asymptomatic patients with bicuspid aortic valve (BAV) and mild aortic valve regurgitation (AR), using cardiac magnetic resonance (CMR) feature tracking and 4D flow imaging. Fifty BAV individuals were retrospectively selected: 15 with mild AR and posterior regurgitation jet (Group-PJ), 15 with regurgitant jet in other directions (Group-nPJ) and 20 with no regurgitation (Controls). CMR protocol included cine steady state free precession (SSFP) sequences and 4D Flow imaging covering the entire left ventricle (LV) cavity and the aortic root. Cine-SSFP images were analyzed to assess LV volumes, longitudinal and circumferential myocardial strain. Circumferential and longitudinal peak diastolic strain rate (PDSR) and peak diastolic velocity (PDV) were reduced in group PJ if compared to group nPJ and control group (PDSR = 1.10 ± 0.2 1/s vs. 1.34 ± 0.5 1/s vs. 1.53 ± 0.3 1/s, p:0.001 and 0.68 ± 0.2 1/s vs. 1.17 ± 0.2 1/s vs. 1.05 ± 0.4 1/s ; p < 0.001, PDV = − 101.6 ± 28.1 deg/s vs. − 201.4 ± 85.9 deg/s vs. − 221.6 ± 67.1 deg/s; p < 0.001 and − 28.1 ± 8 mm/s vs. − 38.9 ± 11.1 mm/s vs. − 43.6 ± 14.3 mm/s, p < 0.001, respectively), whereas no differences have been found in systolic strain values. 4D Flow images (available only in 9 patients) showed deformation of diastolic transmitral streamlines direction in group PJ compared to other groups. In BAV patients with mild AR, the posterior direction of the regurgitant jet may hamper the complete mitral valve opening, disturbing transmitral flow and slowing the LV diastolic filling.

## Introduction

Bicuspid aortic valve (BAV), with a prevalence of 0.5–2% of the general population [1], includes a heterogeneous spectrum of morphological phenotypes, ranging from the symmetric forms (with no raphe) to the asymmetrical forms characterized by partial or complete fusion of the cusps by one or two raphes [2]. BAV is prone to be dysfunctional, as malformed leaflets frequently have reduced opening or undergo to accelerated fibro-calcific degeneration resulting in aortic stenosis. In other cases, the lack of coaptation might cause aortic valve regurgitation (AR), which seems to be more frequent in young male patients [3, 4].

The characteristics of the regurgitant jet and its severity in BAV patients can be highly heterogeneous, as they depend on valve phenotype, cusps asymmetry, aortic root dilation and acquired abnormalities (endocarditis or degenerative fibrocalcific appositions) [5, 6]. Moderate-to-severe AR is associated with left ventricle (LV) diastolic dysfunction [7], ventricular remodeling with eccentric hypertrophy, interstitial fibrosis and, lastly, LV dilation with heart failure (HF) [8]. Conversely, mild AR is commonly asymptomatic and may remain clinically silent for years, even though it is unclear whether there may be early markers predicting disease progression or specific features of clinical relevance.

Specifically, in some cases the regurgitant jet is directed posteriorly against the anterior mitral leaflet, interfering with the valve opening [9, 10], and the hemodynamic effects of this phenomenon have been poorly investigated.

The advanced analysis of cardiac fibers deformability offered by the Cardiovascular Magnetic Resonance (CMR) feature tracking (CMR-FT) technique enables the accurate and reproducible assessment of early modifications in both LV systolic and diastolic function [11–13]. CMR-FT is a post-processing technique applied to cine steady-state free precession (cine-SSFP) sequences, which analyzes myocardial longitudinal and circumferential strain parameters throughout all phases of cardiac cycle [14].

Moreover, the four-dimensional flow (4D Flow) imaging, obtained by acquiring 3D phase contrast images with three-directional velocity encoding, enables to investigate intraventricular blood flow dynamic in normal and pathological conditions [15].

The purpose of our study was to assess the impact of the regurgitant jet direction on left ventricular function and intraventricular hemodynamics in asymptomatic patients with BAV and mild insufficiency.

## Materials and methods

### Study population

We retrospectively selected a cohort of 50 individuals with BAV, who referred our Institution for their periodic follow-up monitoring, between January 2010 and December 2020. CMR exams were selected in order to compose three age- and body surface area (BSA) -matched groups:

- Thirty subjects had mild valve regurgitation: 15/30 with a posteriorly directed jet against the anterior mitral leaflet (*Posterior Jet or PJ Group*) and 15/30 with a regurgitant jet directed to other directions (*Non-Posterior Jet or nPJ Group*).
- Twenty subjects without any regurgitation or with a negligible degree of regurgitation (*Controls*).

Exclusion criteria included: moderate-to-severe aortic valve stenosis, moderate-to-severe aortic regurgitation, prior aortic valve replacement, aortic root enlargement > 45 mm, LV ejection fraction < 50%, incomplete cine-SSFP image dataset or poor image quality (e.g. breathing / movement artefacts, < 20 frames/cardiac cycle, missing long axis views), atrial fibrillation, autoimmune diseases, diabetes, other concurrent known congenital heart disease, genetic syndromes (Turner or Marfan) or cardiovascular disease. We also excluded patients with mitral valve insufficiency or stenosis.

Grading of aortic stenosis and insufficiency was assessed by color-Doppler evaluation at echocardiography.

Before CMR scanning, an anamnestic evaluation and clinical parameters were also collected (blood pressure, cardiac frequency, weight and height, body mass index and body surface area).

### CMR imaging acquisition protocol

CMR images have been acquired on a 1.5 T (Avanto, Siemens Healthcare, Erlangen, Germany; n: 29) and a 3.0 T (Discovery MR750, GE Healthcare, Little Chalfont, United States; n: 21) scanners, both located at the Policlinico Umberto I Hospital of Sapienza University of Rome.

CMR protocol included 2D ECG-gated cine-SSFP images in the short-axis plane covering the entire LV cavity (a stack of 8–10 contiguous slices from the base to the apex) and in two, three and four chambers views, to quantify ventricular size and function and to obtain a complete ventricular function assessment.

A stack of 4 to 6 contiguous cine-SSFP images oriented orthogonal to the longitudinal axis of aortic root covering from aortic annulus to sinotubular junction was acquired in order to analyze valve morphology and function and to measure aortic root diameters.

Cine-SSFP images were performed with a slice thickness of 6 mm for the aortic root and 8 mm for the LV, with no overlap and no gap for the aortic root and an interslice gap of 2 mm for the LV (parameters = Repetition Time: 3.58–3.97 ms; Echo Time: 1.42–1.60 ms; Flip Angle: 45–60°; matrix: 256 × 256; in-plane resolution ranging between 0.73 × 0.73 mm and 1.40 × 1.40 mm; time resolution: 20–25 frames/cardiac cycle). A stack of three 2D phase-contrast (PC) images oriented parallel to aortic root (above, below and at the level of valve annulus) was acquired to analyze transaortic flow velocities (RT: 46.5 ms, ET: 2.44 ms, 20 phases, matrix 176 × 256, FOV 220 × 320 mm^2^, slice thickness 6mm, interslice gap: 2mm in-plane resolution 1.25 × 1.25 mm^2^, Velocity Encoding or VENC: 150 cm/s).

CMR protocol comprehended also the contrast-enhanced MR angiography of the thoracic aorta, by acquiring breath-hold high-resolution 3D Gradient-Echo Flash sequence in oblique sagittal plane before and after contrast administration (0.1 mmol/kg of body weight, Gadobutrol, Bayer) at a flow of 1.5 ml/s; subtracted images were reconstructed to eliminate background signals.

### 4D-flow imaging

Within the total cohort, we selected a sub-group of 9 pts that also performed a 4D-Flow CMR: 3 of them belonging to PJ group, 3 of them to nPJ group and 3 to the control group.

Those patients have been scanned at the 3.0 T magnetic resonance unit using a three-dimensional flow-sensitive gradient-echo pulse sequence, acquired in the oblique-sagittal orientation to encompass thoracic aorta and the entire LV cavity, during free breathing using a respiratory navigator in 20 phases/cardiac cycle.

4D Flow sequences (3dcine_ktarc_WIP package) were performed with the following parameters after gadolinium-based contrast administration: RT = 4.3–5.27 ms, ET = 2.02–2.15 ms, FA = 10–14°, voxel size = 1.48 × 1.48 × 1.8 mm^3^; VENC range had been properly tailored as the lowest value avoiding aliasing, generally 150 cm/s; acquisition time of the 4D Flow sequence ranged from 15 to 20 min based on the heart rate and breathing rate (the duration of the entire CMR protocol, including 4D Flow, has been about 40–45 min).

### Image analysis

BAV type classification was performed according to Sievers’ classification [2]. Maximum transverse aortic root diameters have been measured at the aortic annulus, the Sinuses of Valsalva, the sinotubular junction and ascending aorta.

Two operators in consensus with 14- and 18- year-old experience in CMR imaging (N.G. and M.F., respectively) visually evaluated the presence and the direction of diastolic aortic valve regurgitant jet.

Aortic trans-valvular flow was, then, quantitatively measured using PC sequences (2D and/or 3D when present) to assess the peak of maximum velocity (Vpeak) and aortic valve area, in order to classify the aortic stenosis degree, and the regurgitation fraction (RF), defined as the ratio between regurgitant volume and stroke volume. We enrolled only patients with a aortic valve Vpeak < 250 cm/s and RF < 29%, whereas in control group the RF was lower than 5%. Trans-valvular aortic flow was assessed also on 4D Flow images, when acquired. In case of discordance between measurements obtained by 2D PC and 4D Flow datasets, the highest values were considered. All patients satisfied the inclusion criteria both when assessed by 2D PC and 4D Flow images.

LV volumes measurements were performed using a dedicated software (cvi42® v.5.3, Circle Cardiovascular Imaging, Calgary, Canada). LV endocardial and epicardial contours were manually traced on short-axis cine-SSFP images in all the phases of cardiac cycle [16]. Analysis of the volume-time curves of the LV cavity has been performed to evaluate the peak filling rate (PFR) and the time-to-peak filling rate (TTPFR). The size of left atrium (LA) has been measured in all patients as the LA area on the cine-SSFP image acquired on four-chamber view at the end-systole, on the frame just prior to mitral valve opening by tracing the LA inner border, excluding the area under the mitral valve annulus and the inlet of the pulmonary veins.

The 2D tissue tracking analysis was assessed using mid-ventricular short-axis, vertical- and horizontal long-axis cine-SSFP images. The epicardial and endocardial contours were drawn at end-diastolic phases. Our software (Tissue Tracking module) was able to generate a pixel-intensity map for a small region of the myocardium on cine-SSFP images, using algorithms to identify the most similar patterns of pixel-intensity on images from all subsequent images in the cardiac cycle to “track” a specific point in the myocardium. Myocardial tracking was visually reviewed and the contouring errors in tracking the myocardial borders were corrected before repetition of the analysis.

We measured circumferential (c-) and longitudinal (l-) components of the different LV strain parameters in order to detect any alteration in myocardial fibers deformability during the systolic and diastolic relaxation phases: peak systolic strain (%), peak systolic strain rate (%/s), time to peak strain (s), peak diastolic strain rate (%/s), peak diastolic velocity (mm/s).

Circumferential strain values were calculated as the mean of values measured on three mid-ventricular short axis views in order to warrant adequate reproducibility of measurements and representativeness of myocardial contractile dysfunction.

Longitudinal strain values have been obtained as the mean of strain values measured on vertical- and horizontal long-axis.

### 4D flow image analysis

4D Flow data have been automatically processed by using a dedicated commercially-available software (4D Flow module, cvi42® v.5.9.2, Circle Cardiovascular Imaging, Calgary, Canada) in order to assess intraventricular instantaneous velocity vectors and to generate path-lines and streamlines dynamic graphs. 4D Flow data were automatically processed through prefiltering of velocity components to correct for eddy current errors and aliasing and to decrease signal noise. The path-lines and streamline maps have been elaborated using ventricular long axis projections on the background (horizontal, vertical and three-chamber) and the most representative time-frame of mid-diastolic phase was selected in order to identify the predominant orientation axis of transmitral diastolic flow.

We also analyzed the left intraventricular flow using path-lines in order to understand whether the aortic valve regurgitation had a macroscopic impact on flow pattern and mitral valve kinetics.

### Statistical analysis

All the data are presented as counts and percentages for categorical data and means ± standard deviation for continuous data, whose distributions were tested for normality using Shapiro-Wilk test. CMR and clinical parameters comparisons were undertaken using the one-way analysis of variance (ANOVA) with Bonferroni’s post-hoc test for normally distributed values or Kruskal-Wallis test with Bonferroni’s post-hoc test for non-normal distributions. For categorical data the Chi-square (X^2^) test was performed. All the tests were 2-tailed, and only P values < 0.05 were considered statistically significant. Analysis was performed using SPSS software version 26.0 (IBM).

## Results

### Patient characteristics

Our population was mainly composed of adolescent and young adult (age: 24.4 ± 11 years-old), with a prevalence of male gender (n, male:female = 34:16).

No significant differences have been found among three groups in mean age, BSA and body mass index (BMI).

**Fig. 1:**
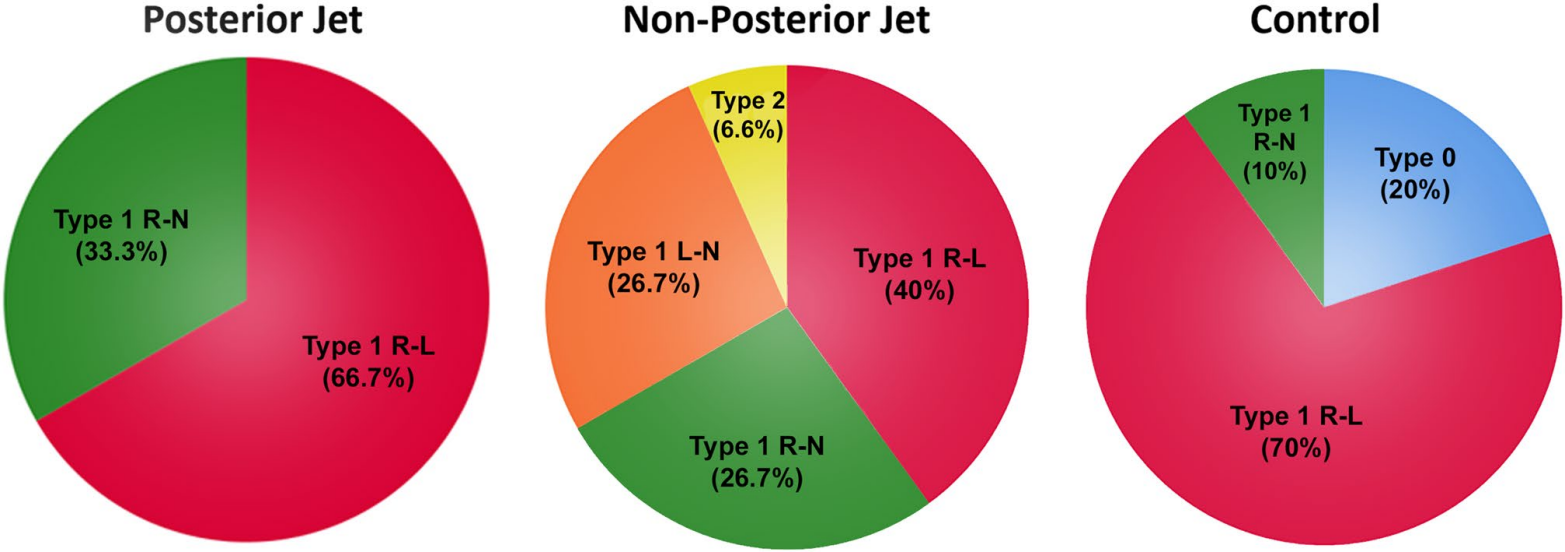
BAV phenotypes according to Sievers classification. BAV types percentages according to Sievers classification among “Posterior jet” (PJ) group, “Non-posterior jet” (nPJ) group and controls. Type 1 R–L resulted to be the more represented (66.7% in PJ; 40% in nPJ and 70% in controls). Type 1 R–N was the second most frequent phenotype (33.3% in PJ; 26.7% in nPJ and 10% in controls). PJ group was only composed by type 1 R–L and R–N; type 1 L–N and type 2 were only found in nPJ group (26.7 and 6.7%, respectively); type 0 was only present among controls (20%). *BAV* Bicuspid aortic valve; *L–N* left – non coronary cusps; *R–L* right-left coronary cusps; *R–N* right – non coronary cusps

The only BAV phenotypes represented in PJ group were type 1 R–L (67%) and type 1 R–N (33%) (Fig. 1).

Population’s characteristics and parameters are reported in Table 1.

**Table 1:** Clinical Characteristics, Aortic Valve features, aortic root diameters

Characteristics	Posterior jet n:15	Non posterior jet n. 15	Controls n: 20	p
Males	11 (83.3)	8 (58.3)	15 (75)	**0.011**
Age, years	25.7 ± 11.1	22.9 ± 10.5	25.5 ± 12.1	0.363
BSA, m^2^	1.77 ± 0.2	1.77 ± 0.4	1.76 ± 0.2	0.875
BMI, kg/m^2^	23.2 ± 3.1	23.04 ± 3.9	23.5 ± 2.5	0.866
Systolic BP, mmHg	116.8 ± 7.3	123.4 ± 8.9	121.2 ± 8.4	0.097
Diastolic BP, mmHg	61.7 ± 4.5	62.7 ± 7.2	64.8 ± 7.8	0.509
Heart Rate, beats/min	69 ± 3.1	70 ± 2.6	67 ± 3.7	0.941
Hypertension	0 (0)	1 (6.7)	2 (10)	0.464
Smoking	5 (33.3)	3 (20)	5 (25)	0.701
Bicuspid aortic valve morphology (sievers classification)				
TYPE 0 (no raphe)	0	0	4 (20)	–
TYPE 1 R-L	10 (66.7)	6 (40)	14 (70)	–
TYPE 1 R-N	5 (33.3)	4 (26.7)	2 (10)	–
TYPE 1 L-N	0	4 (26.7)	0	–
TYPE 2 (2 raphes)	0	1 (6.7)	0	–
Aortic valve stenosis				
No	13 (86.6)	12 (80)	13 (65)	–
Minimal	1 (6.7)	2 (13.3)	4 (20)	–
Mild	1 (6.7)	1(6.7)	3 (15)	–
Aortic valve insufficiency				
No	0	0	12	–
Minimal	0	0	8	–
Mild	15	15	0	–
Regurgitation fraction (%)	13.4 ± 9.4	9.2 ± 8.4	3.5 ± 3.2	**< 0.001**
Aortic root diameters				
Aortic annulus diameter, mm	27.64 ± 2.8	25.67 ± 3.2	24.3 ± 2.9	**0.003**
Aortic sinus diameter, mm	33.3 ± 3.7	32 ± 3.3	33.8 ± 3.9	0.411
Sino-tubular junction, mm	30 ± 4.5	28.3 ± 1.7	28.3 ± 3.2	0.384
Ascending aorta diameter, mm	33.5 ± 6.2	32.3 ± 5.7	33.4 ± 3.4	0.444

Values are expressed as mean ± SD or n (%). P values in bold indicate a p value < 0.05

*BSA* body surface area,* BMI* body mass index,* BP* blood pressure,* L–N* left–non coronary cusps,* R–L* right-left coronary cusps,* R–N* right – non coronary cusps

### LV volumes and strain parameters

LV stroke volume, ejection fraction and myocardial mass did not show any significant differences between the three groups as well as systolic strain parameters (Table 2).

**Table 2:** Ventricular Volumes and LV strain parameters

CMR features	Posterior jet n. 15	Non-posterior jet n:15	Controls n: 20	p
Left atrium area, cm²	19.3 ± 2.0	17.6 ± 2.3	19.7 ± 3.4	0.105
LV transversal end-diastolic diameter, mm	55.7 ± 6	53.1 ± 5	53.2 ± 3.9	0.295
Interventricular Septum, mm	9.53 ± 1.8	9.32 ± 2.0	9.92 ± 1.3	0.575
Ventricular Volumes				
LV-EDVi, ml/m^2^	97.7 ± 15.7	85.3 ± 11.7	79.3 ± 13.6	**0.001**
LV-ESVi, ml/m^2^	41.2 ± 11.8	34.3 ± 5.6	31.2 ± 7.3	**0.002**
LV EF, %	57.2 ± 5.8	60.2 ± 3.5	60.5 ± 5.5	0.099
LV-SVi, ml/m^2^	50.3 ± 8.3	50.6 ± 8.5	46.6 ± 8.3	0.276
LV-Mi, g/m^2^	61.6 ± 8.4	57.7 ± 18.4	57.4 ± 8.2	0.170
LV peak filling rate, mL/s	406.5 ± 58.5	394.5 ± 146	358 ± 100	0.147
LV time to peak filling rate, ms	477.9 ± 19.1	447.7 ± 22.8	412.1 ± 37.5	**0.001**
RV-EDVi, ml/m^2^	93.3 ± 15.2	93.5 ± 13.8	92.2 ± 15.8	0.684
RV-ESVi, ml/m^2^	50.6 ± 11.1	47.2 ± 10.0	45.2 ± 10.4	0.172
RV-EF, %	47.9 ± 5.5	50.0 ± 5.7	51.1 ± 5.3	0.206
RV-SVi, ml/m^2^	45.8 ± 7.2	46.2 ± 6.5	47.0 ± 7.4	0.799
LV myocardial strain parameters				
Peak systolic strain—circumferential, %	− 18.42 ± 2.5	− 17.99 ± 2.4	− 18.84 ± 3.7	0.688
Peak systolic strain—longitudinal, %	− 15.91 ± 2.9	− 16.01 ± 2.6	− 17.85 ± 3.8	0.286
Peak systolic strain rate—circumferential, 1/s	− 1.07 ± 0.13	− 1.02 ± 0.12	− 0.95 ± 0.24	0.106
Peak systolic strain rate—longitudinal, 1/s	− 0.97 ± 0.19	− 0.99 ± 0.18	− 0.89 ± 0.21	0.091
Time to peak systolic strain—circumferential, ms	289.9 ± 38.63	292.9 ± 55.8	275.4 ± 108.8	0.922
Time to peak systolic strain—longitudinal, ms	304.6 ± 64.9	284.6 ± 34.4	284.2 ± 100.1	0.778
Peak diastolic strain rate—circumferential, 1/s	1.10 ± 0.2	1.34 ± 0.5	1.53 ± 0.3	**0.001**
Peak diastolic strain rate—longitudinal, 1/s	0.68 ± 0.2	1.17 ± 0.2	1.05 ± 0.4	**< 0.001**
Peak diastolic velocity—circumferential, deg/s	− 101.6 ± 28.1	− 201.4 ± 85.9	− 221.6 ± 67.1	**< 0.001**
Peak diastolic velocity—longitudinal, mm/s	− 28.1 ± 8	− 38.9 ± 11.1	− 43.6 ± 14.3	**< 0.001**

Values are expressed as mean ± SD or n (%). P values in bold indicate a p value < 0.05

*LV* left ventricle,* LV-EDVi* indexed left ventricular end-diastolic volume,* LV-EF* left ventricular ejection fraction,* LV-ESVi* indexed left ventricular end-systolic volume,* LV-SVi* indexed left ventricular stroke volume,* LV-Mi* indexed left ventricular myocardial mass,* RV* right ventricle,* RV-EDVi* indexed right ventricular end-diastolic volume,* RV-EF* right ventricular ejection fraction,* RV-ESVi* indexed right ventricular end-systolic volume,* RV-SVi* indexed right ventricular stroke volume,* SD* standard deviation

Circumferential peak diastolic strain rate was decreased in PJ group if compared to nPJ group and the control group (1.10 ± 0.2 1/s vs. 1.34 ± 0.5 1/s vs. 1.53 ± 0.3 1/s, p: 0.001). The same result was found for the longitudinal peak diastolic strain rate (0.68 ± 0.2 1/s vs. 1.17 ± 0.2 1/s vs. and 1.05 ± 0.4 1/s; p < 0.001), that was significantly lower in PJ group as compared to nPJ group and control group.

**Fig. 2:**
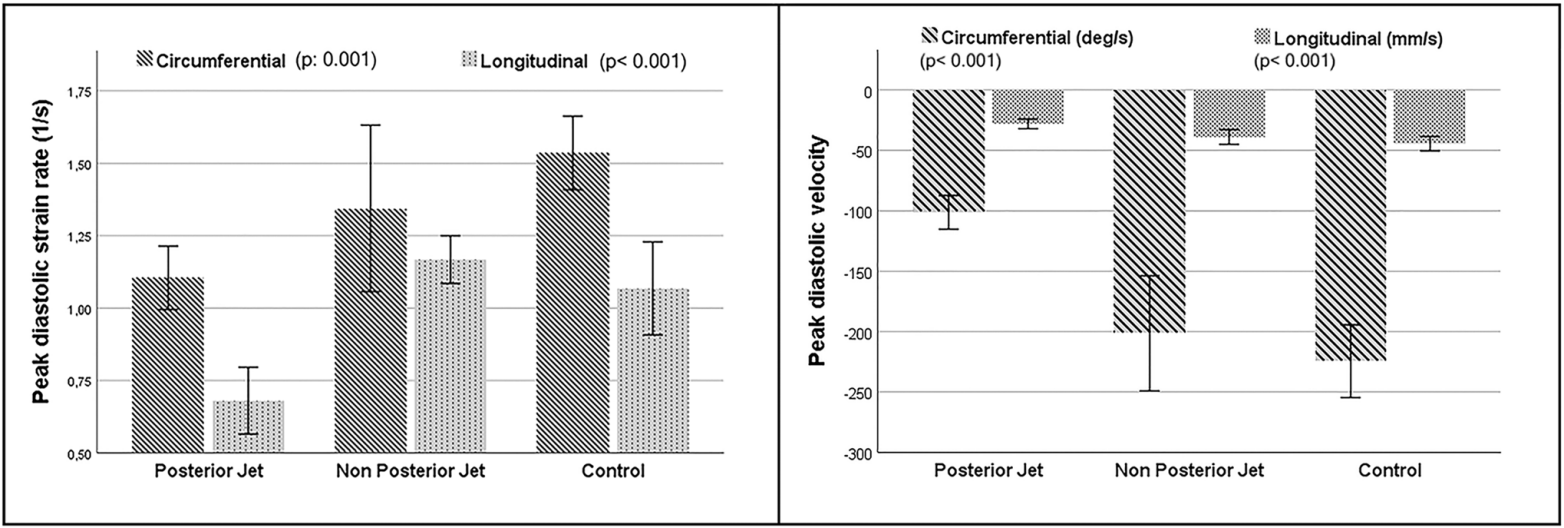
Histograms of diastolic strain parameters by feature tracking technique. Comparison between PJ, nPJ and control groups as regards peak diastolic strain rate (on the left) and peak diastolic velocity (on the right). The top of the histogram represents the mean value; the error bar represents the confidence interval (95%). *PJ* posterior jet; *nPJ* non-posterior jet

LV strain results were associated to a decreased circumferential peak diastolic velocity (− 101.6 ± 28.1 deg/s vs. − 201.4 ± 85.9 deg/s vs. − 221.6 ± 67.1 deg/s; p < 0.001) and longitudinal peak diastolic velocity (− 28.1 ± 8 mm/s vs. − 38.9 ± 11.1 mm/s vs. − 43.6 ± 14.3 mm/s, p < 0.001) in PJ group, as compared to the other two groups (Fig. 2).

No differences have been detected between the groups, regarding the PFR and LA size, even though a negative correlation between the LA size and the longitudinal diastolic strain rate (r: − 0.69, p: 0.004) was found in PJ group. Notably, PJ group showed the longest TTPFR value (477.9 ± 19.1 ms vs. 447.7 ± 22.8 ms vs. 412.1 ± 37.5 ms, p:0.001), reflecting an elongation of the early diastolic phase.

PJ group showed greater aortic annulus diameters than the control group (27.64 ± 2.8 mm vs. 24.3 ± 2.9 mm, p:0.002), with no significant differences in the comparisons between PJ Vs nPJ group (p: 0.164) and nPJ Vs control group (p: 0.383).

### 4D flow imaging

The visual analysis of path-lines dynamic graphs demonstrated an angulation of the predominant axial orientation of the trans-mitral diastolic flow as compared to the LV longitudinal axis in all PJ subjects. Whereas nPJ subjects and controls showed a complete alignment of the two axes in all cases.

**Fig. 3:**
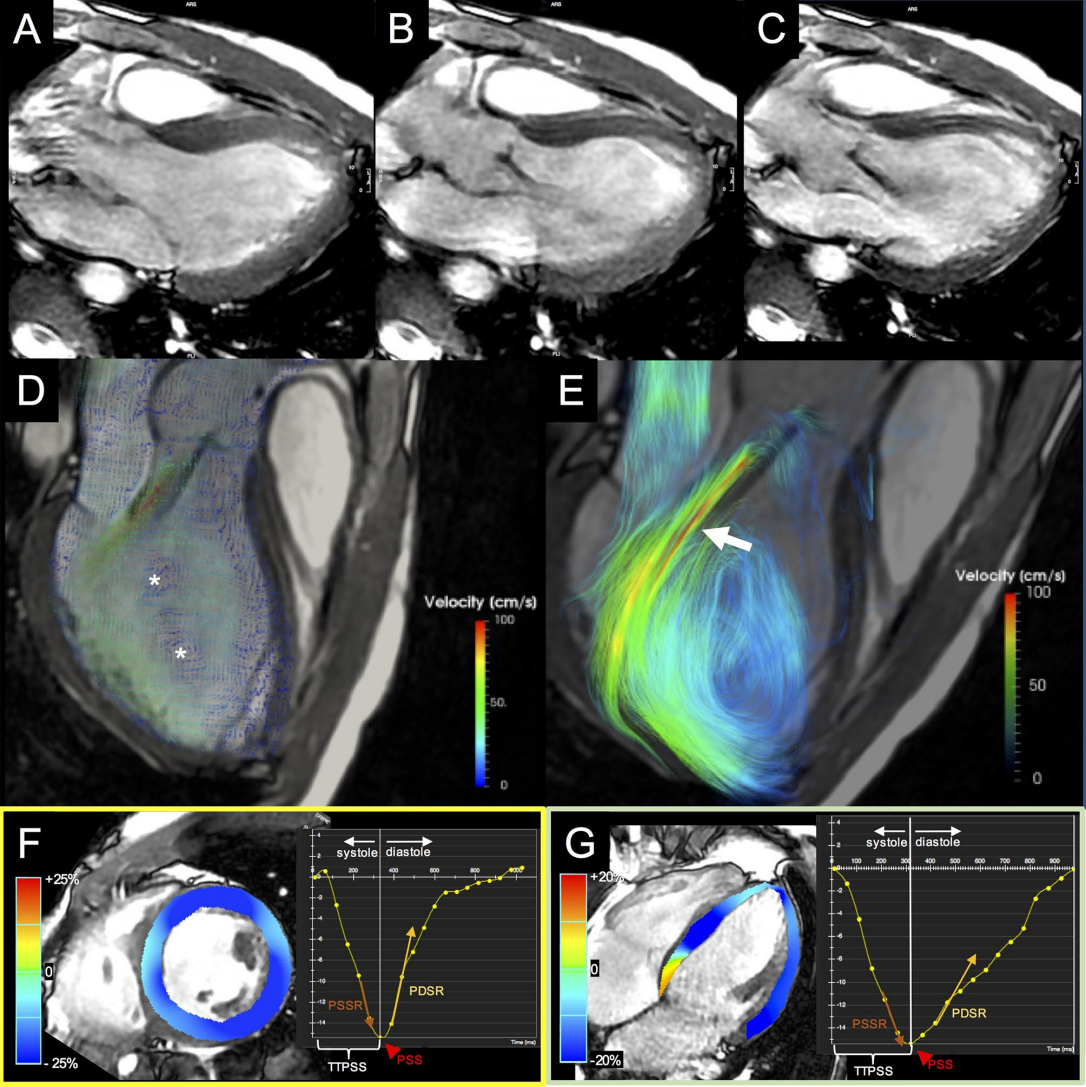
BAV patient with aortic regurgitation and posterior jet. Set of three chamber cine-SSFP images acquired during **A** systolic, **B** mid-diastolic and **C** end-diastolic phase, shows aortic valve leaflets movement over the entire cardiac cycle, with the regurgitation jet impacting the anterior mitral valve leaflet. 4D Flow color encoded vector map (**D**) and streamlines (**E**) at the mid diastole demonstrate the abnormal intracavitary flow with generation of large organized mid-ventricular vortexes (asterisks) caused by the interference from an eccentric and angulated regurgitation jet (arrow) on the transmitral diastolic flow. Analysis of circumferential and longitudinal strain was respectively performed on cine-SSFP images acquired on short-axis (**F**) and horizontal long-axis (**G**) views. Systolic and diastolic strain values have been assessed as peak systolic strain (PSS, red arrowheads), peak systolic strain rate (PSSR, orange arrows), time-to-peak systolic strain (TTPSS, white brackets) and peak diastolic strain rate (PDSR, yellow arrows). *SSFP* Steady state free precession

The aortic regurgitant jet was depicted by path-lines maps in all PJ cases and 1/3 nPJ case, while in 2/3 nPJ cases it was not clearly distinguishable. In all three PJ subjects, the regurgitant jet clearly hampered the opening of the mitral valve, causing an abnormal angulation and eccentricity of the transmitral flow (Fig. 3).

## Discussion

Our study demonstrated that, in BAV patients, the posterior direction of regurgitant jet may cause LV diastolic dysfunction, even in patients with mild AR. Jet impacting on the anterior mitral leaflet was associated to a significant reduction in circumferential and longitudinal diastolic strain rate and peak diastolic velocity as compared to AR patients with non-impacting jet and controls, with no differences in myocardial systolic strain parameters. Comparing our results with those assessed in healthy subjects reported in other studies [17–19], we found that systolic strain values were within normal range in all groups [17] whereas in PJ group the diastolic strain values were at the lower limits of normal range for circumferential component and markedly reduced for longitudinal.

To our knowledge, this is the first study that correlates AR jet direction to LV function using CMR.

Although our study population was mainly composed by young and asymptomatic patients, CMR was able to detect the presence of a subtle and clinically silent ventricular function alteration, represented by impaired diastolic function.

After the systolic contraction and the closure of the aortic valve (proto-diastolic phase), the inversion of the pressure gradient between the left atrium and ventricle and relaxation of the ventricular wall cause the opening of the mitral valve, allowing the rapid filling of the ventricle (rapid isotonic diastole).

When posteriorly directed, the eccentric regurgitant jet may hinder normal anterior mitral leaflet mobility causing a fluttering of the valve leaflet and delayed opening [10], resulting in an impairment of the early filling.

Indeed, in our study, PJ group had a delay in peak filling (increase TTPFR), even there were no differences in terms of PFR among three groups.

In cases of moderate-to-severe chronic AR this mechanism may induce asymmetrical mitral valve (MV) remodeling [9], with enlargement and rarely perforation of the anterior leaflet, causing severe mitral insufficiency [20–22].

Diastolic dysfunction, defined as an increased resistance to filling by the LV [23], can be classified in three grades [24] and can be associated to a simple increase in LV end-diastolic pressure and/or to an elevated mean left atrial pressure (which results in a pulmonary venous hypertension, pulmonary congestion and dyspnea) [24].

In our population, PJ patients had both increased end diastolic and systolic LV volumes as compared to nPJ and controls, whereas there were no differences between nPJ and controls, highlighting how the direction of the jet can affect the ventricular filling volumes, even if the regurgitation is not severe enough to cause volume overload.

The early identification of subclinical diastolic dysfunction in BAV patients with preserved ejection fraction and mild AR may be benefical for improving their risk stratification and predict long-term outcome.

In fact, diastolic dysfunction represents the first altered parameter in the progressive process that may finally result in HF. It is known that the 5-year mortality rate for individuals with HF, preserved ejection fraction and diastolic dysfunction, ranges between 55 and 74% [25].

Moreover, BAV condition *per se* leads to a greater incidence of cardiovascular complications as compared to the general population (dilation or dissection of the thoracic aorta, aortic valve stenosis or insufficiency, endocarditis and myocardial ischemia) [25–28]. Furthermore, PJ group showed increased aortic annulus diameters as compared to the other groups, suggesting the possibility of additional pathological mechanisms related to the jet eccentricity and cusps asymmetry.

The use of CMR-FT to evaluate LV diastolic impairment has already been performed in asymptomatic patients with BAV and preserved ejection fraction [14], evidencing an alteration in diastolic strain parameters in BAV subjects as compared to control groups [14]. Other studies revealed that CMR-FT myocardial diastolic strain analysis was able to predict adverse outcomes in patients with hypertrophic cardiomyopathy or atherosclerosis [28, 29].

Finally, the use of 4D Flow imaging technique offered a deeper insight on the hemodynamic consequences on intraventricular flows of different patterns of regurgitation jet.

**Fig. 4:**
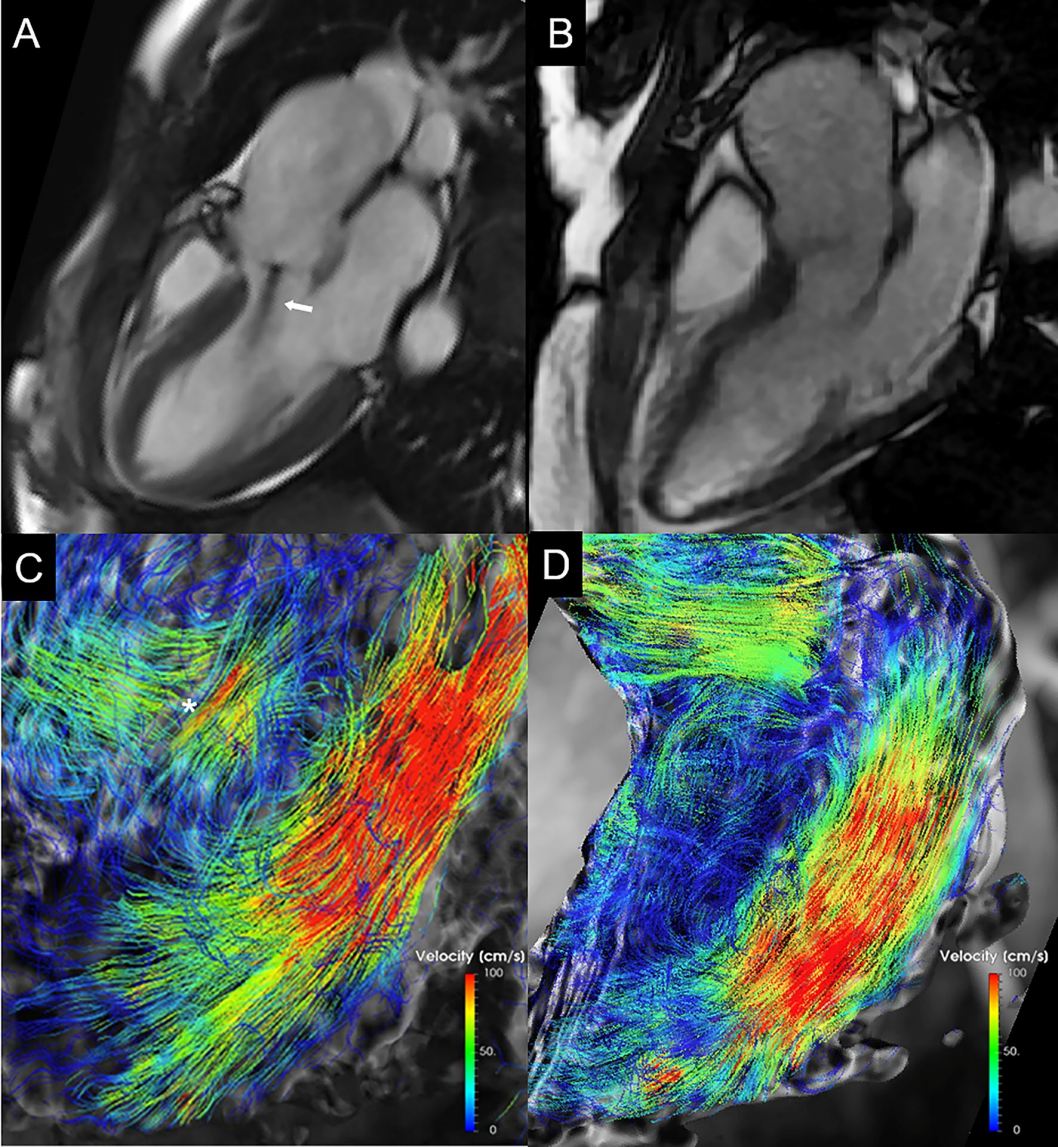
BAV patients with (non-posterior jet) and without aortic regurgitation. Three chamber cine-SSFP images acquired during mid diastolic phase in BAV patient with aortic valve regurgitation and jet non impacting the valve leaflet (**A**, arrow) and in BAV patient with no regurgitation (**B**). The corresponding 4D Flow color encoded streamlines images, matching the same three-chamber views, demonstrated similar organized laminar diastolic transvalvular flow, both in patient with (**C**) and without aortic regurgitation (**D**) with predominant streamline orientation along the LV longitudinal axis. The asterisk marks the regurgitant flow within LV outflow tract just under the aortic valve. *BAV* bicuspid aortic valve; *LV* left ventricle; *SSFP* steady state free precession

As explained in in-vitro studies conducted on insufficient aortic valves, the presence of a regurgitation jet generates an anticlockwise intraventricular vortex which hampers LV filling, as the degree of regurgitation increases, interacting with the clockwise vortex coming from the mitral valve [30, 31]. We can assume that this mechanism is even more emphasized by the posterior direction of the regurgitation jet, as opposed to non-posterior, which does not seem to interfere with intraventricular hemodynamics in mild AR (Fig. 4).

Indeed, according to our 4D Flow imaging analysis, in PJ group the AR jet deviates the transmitral inflow from the normal direction towards the inferolateral wall, likely increasing local wall stress and influencing intraventricular vortex formation. Conversely, in nPJ group the transmitral diastolic flow direction was preserved with main orientation parallel to the LV longitudinal axis.

The proper vortices generation seems to have a crucial role in the dynamic balance between rotating blood and myocardial tissue contractile activity. Maladaptive intracardiac vortex dynamics may modulate the progressive remodelling of the left ventricle towards cardiac dysfunction (32).

Further 4D flow imaging studies could offer new perspectives to better understand the effect of aortic valve abnormalities not only on downstream flow (33), but also within the ventricular cavity.

Actually, AR is classified by a combination of clinical signs, LV function and size, and aortic regurgitation degree [32]. However, it is poorly known whether specific features (e.g. valve morphology, regurgitation jet pattern) may promote diastolic dysfunction and LV remodeling, even in mild-to-moderate forms.

In our analysis, the presence of posterior jet was most frequently related to the type I L–R configuration where the conjoined cusp was often responsible for the prolapse causing the posteriorly directed jet (it is not a case that, although our study group was rather small, no type 0 or type I L–N were found in the PJ group).

Moreover, the type I L–R configuration is the most frequent form of BAV phenotype, therefore a posterior jet is also a relatively frequent finding.

Finally, given that the AR recurrence after BAV repair is not rare, surgical planning should consider those phenotypes associated to greater risk of residual posteriorly directed jet.

Another aspect of interest would be the evaluation of jet direction effects on intraventricular hemodynamics, during physical or pharmacological stress. Some studies conducted on BAV athletes with mild AR noticed an increase in LV diameters in BAV as compared to tricuspid aortic valve subjects, but LV volumes were within the normal range [33]. How the hemodynamic adaptation to sport activity can influence regurgitation jet direction and, eventually, intracavitary vortexes formation still needs to be investigated.

### Study limitations

Our study could be limited by the relatively small sample size of the population, which did not allow the analysis of correlation with the different bicuspid phenotypes. In addition, the low number of individuals who underwent 4D Flow imaging prevented us to elaborate quantitative comparison analysis on 4D Flow data.

We also recognize that the lack of follow-up data did not allow defining the long-term clinical relevance of these phenomena in terms of valve disease progression, ventricular remodeling and ventricular function deterioration. Furthermore, this study did not include other CMR features able to assess the diastolic dysfunction, such as the assessment of transmitral valve flow or through the pulmonary veins [34, 35], which would offer a confirmation of the aforementioned findings and a deeper understanding of the mechanisms involved. However, mitral/pulmonary venous flow assessment requires additional sequences (2D or 3D-PC) that which are not part of our routine protocol and have not been systematically acquired in the whole population, therefore that information is no longer obtainable retrospectively.

## Conclusions

In conclusion, the posterior regurgitation jet direction is associate to the presence of a subclinical diastolic dysfunction in BAV patients with mild AR and preserved ejection fraction. The identification of an early stage of diastolic dysfunction with strain imaging could improve the future management of those patients and prevent the worsening of their clinical condition.
